# Supplementary figures and images for: Method for simultaneous tracking of thousands of unlabeled cells within a transparent 3D matrix
Source: PLoS One. 2022 Jun 24;17(6):e0270456. doi: 10.1371/journal.pone.0270456 (PMC9232129; doi:10.1371/journal.pone.0270456)

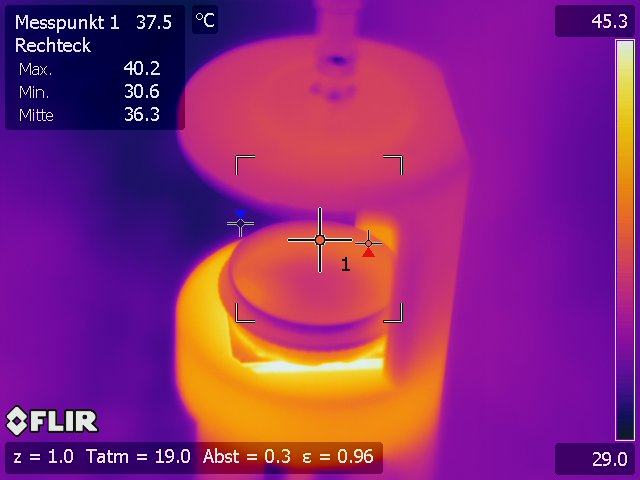

Supplement: S2 Fig — Infrared image of our holographic setup right after a time-lapse recording of 60 min inside of an incubator at 37 degrees air temperature (IR camera FLIR P660). Four measuring points are displayed in the upper left box; the top reading of 37.5 degrees refers to the large crosshair and refers to the highest temperatures found within the petri dish. Therefore, the cells are not subjected to temperatures significantly higher than 37°C. (TIF) [file pone.0270456.s002.tif]

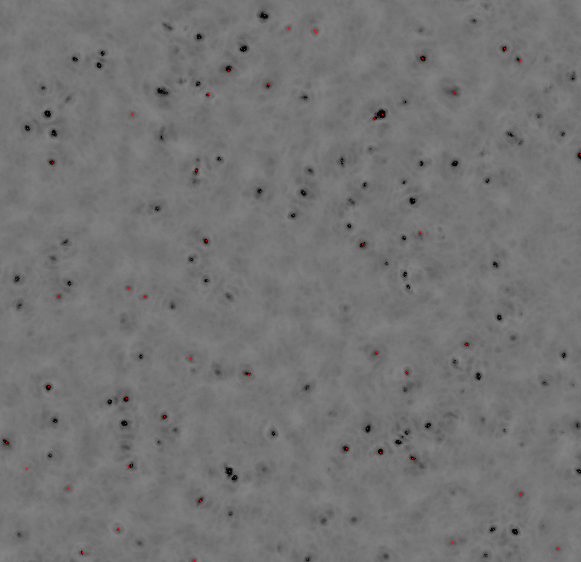

Supplement: S1 Dataset — Ground truth for evaluation of the tracking algorithm. A subset of 50 frames (about 3.5 min) and size 581x562 pixel (650x630 um) was manually annotated in the phase shift minimum projections of a time-lapse recording of neutrophils (stimulation 100 ng/ml IL-8). For results of benchmarking our algorithm see S4 Fig. (ZIP) [file pone.0270456.s007.zip › 000101.bmp]

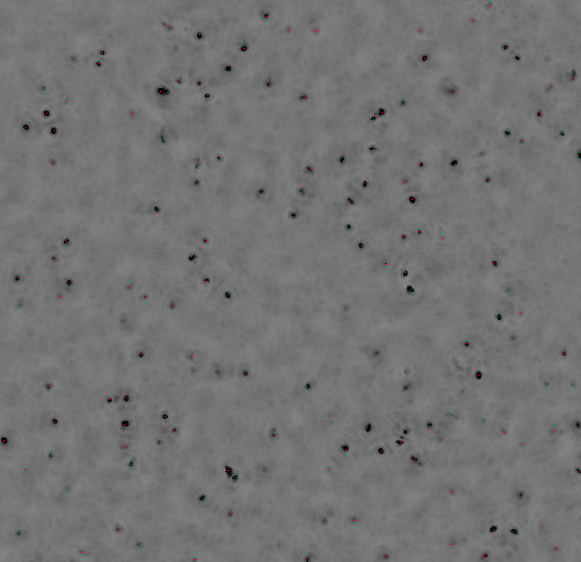

Supplement: S1 Dataset — Ground truth for evaluation of the tracking algorithm. A subset of 50 frames (about 3.5 min) and size 581x562 pixel (650x630 um) was manually annotated in the phase shift minimum projections of a time-lapse recording of neutrophils (stimulation 100 ng/ml IL-8). For results of benchmarking our algorithm see S4 Fig. (ZIP) [file pone.0270456.s007.zip › 000102.bmp]

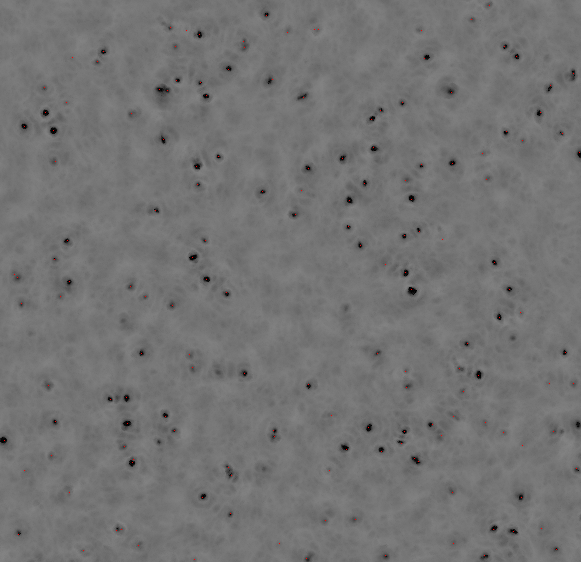

Supplement: S1 Dataset — Ground truth for evaluation of the tracking algorithm. A subset of 50 frames (about 3.5 min) and size 581x562 pixel (650x630 um) was manually annotated in the phase shift minimum projections of a time-lapse recording of neutrophils (stimulation 100 ng/ml IL-8). For results of benchmarking our algorithm see S4 Fig. (ZIP) [file pone.0270456.s007.zip › 000103.bmp]

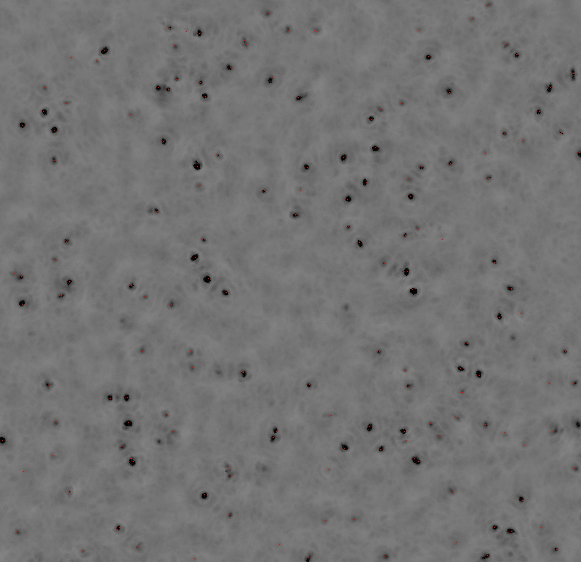

Supplement: S1 Dataset — Ground truth for evaluation of the tracking algorithm. A subset of 50 frames (about 3.5 min) and size 581x562 pixel (650x630 um) was manually annotated in the phase shift minimum projections of a time-lapse recording of neutrophils (stimulation 100 ng/ml IL-8). For results of benchmarking our algorithm see S4 Fig. (ZIP) [file pone.0270456.s007.zip › 000104.bmp]

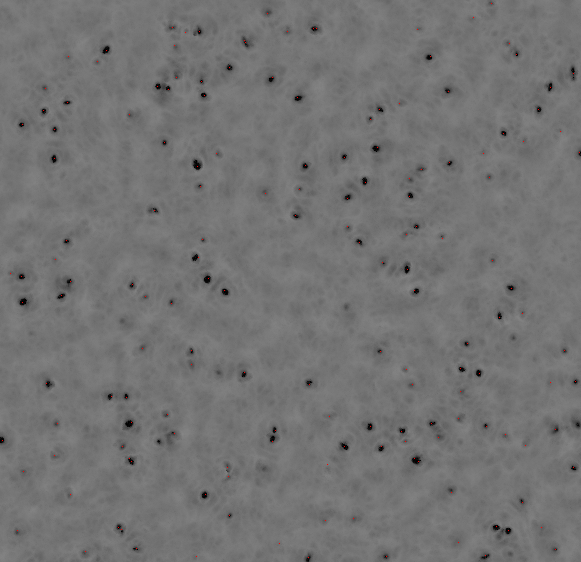

Supplement: S1 Dataset — Ground truth for evaluation of the tracking algorithm. A subset of 50 frames (about 3.5 min) and size 581x562 pixel (650x630 um) was manually annotated in the phase shift minimum projections of a time-lapse recording of neutrophils (stimulation 100 ng/ml IL-8). For results of benchmarking our algorithm see S4 Fig. (ZIP) [file pone.0270456.s007.zip › 000105.bmp]

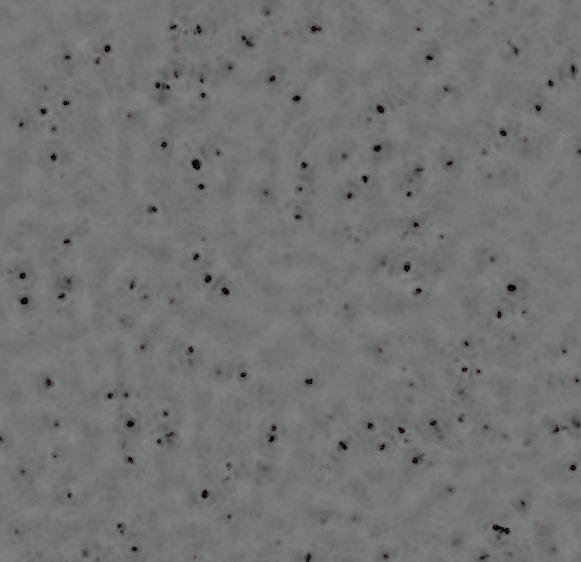

Supplement: S1 Dataset — Ground truth for evaluation of the tracking algorithm. A subset of 50 frames (about 3.5 min) and size 581x562 pixel (650x630 um) was manually annotated in the phase shift minimum projections of a time-lapse recording of neutrophils (stimulation 100 ng/ml IL-8). For results of benchmarking our algorithm see S4 Fig. (ZIP) [file pone.0270456.s007.zip › 000106.bmp]

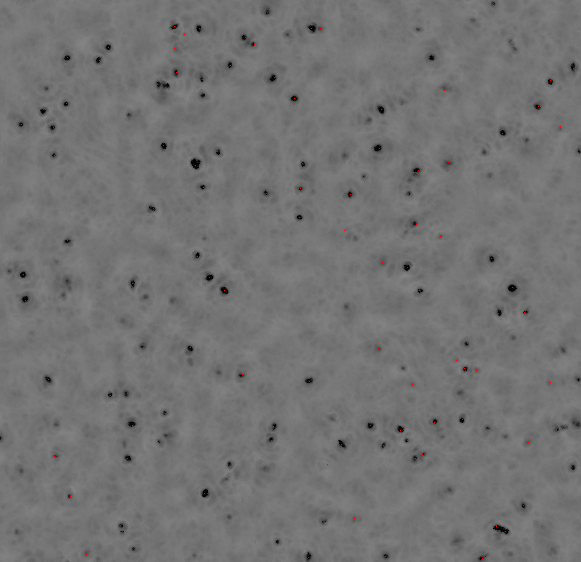

Supplement: S1 Dataset — Ground truth for evaluation of the tracking algorithm. A subset of 50 frames (about 3.5 min) and size 581x562 pixel (650x630 um) was manually annotated in the phase shift minimum projections of a time-lapse recording of neutrophils (stimulation 100 ng/ml IL-8). For results of benchmarking our algorithm see S4 Fig. (ZIP) [file pone.0270456.s007.zip › 000107.bmp]

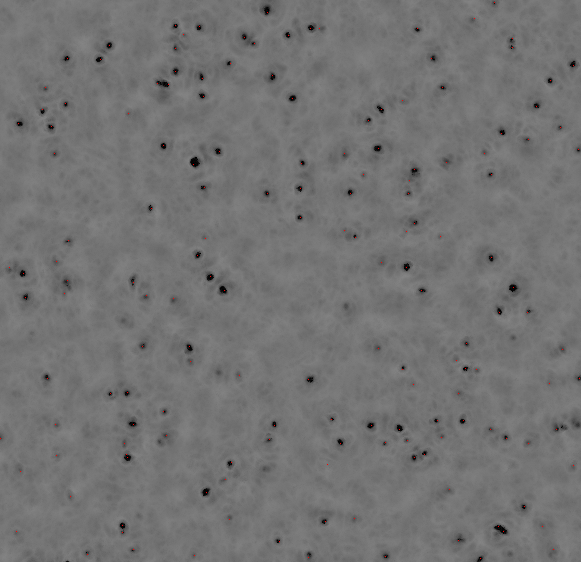

Supplement: S1 Dataset — Ground truth for evaluation of the tracking algorithm. A subset of 50 frames (about 3.5 min) and size 581x562 pixel (650x630 um) was manually annotated in the phase shift minimum projections of a time-lapse recording of neutrophils (stimulation 100 ng/ml IL-8). For results of benchmarking our algorithm see S4 Fig. (ZIP) [file pone.0270456.s007.zip › 000108.bmp]

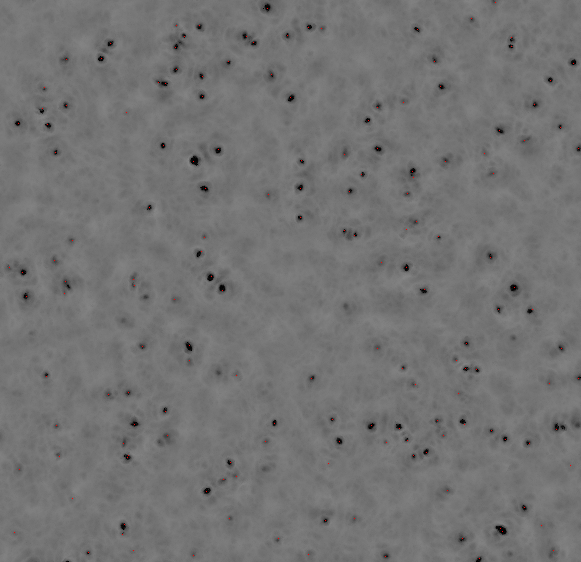

Supplement: S1 Dataset — Ground truth for evaluation of the tracking algorithm. A subset of 50 frames (about 3.5 min) and size 581x562 pixel (650x630 um) was manually annotated in the phase shift minimum projections of a time-lapse recording of neutrophils (stimulation 100 ng/ml IL-8). For results of benchmarking our algorithm see S4 Fig. (ZIP) [file pone.0270456.s007.zip › 000109.bmp]

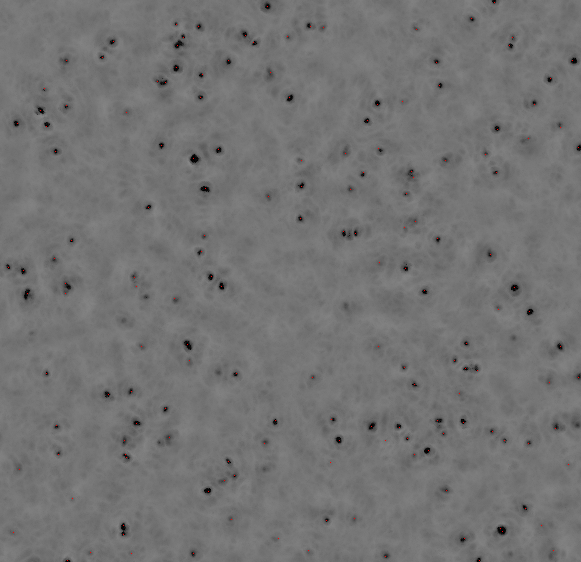

Supplement: S1 Dataset — Ground truth for evaluation of the tracking algorithm. A subset of 50 frames (about 3.5 min) and size 581x562 pixel (650x630 um) was manually annotated in the phase shift minimum projections of a time-lapse recording of neutrophils (stimulation 100 ng/ml IL-8). For results of benchmarking our algorithm see S4 Fig. (ZIP) [file pone.0270456.s007.zip › 000110.bmp]

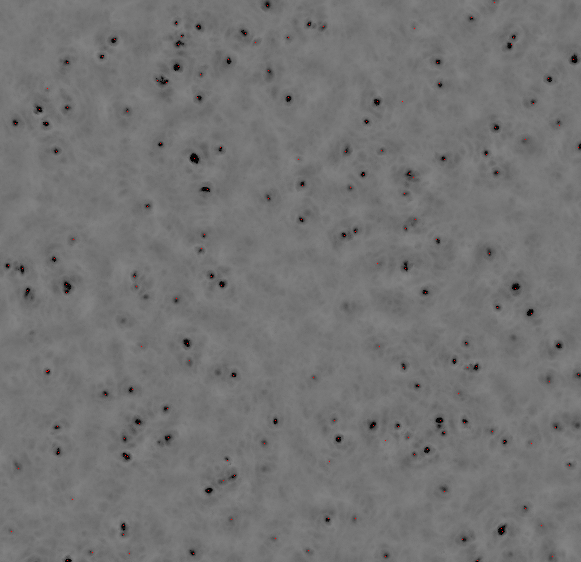

Supplement: S1 Dataset — Ground truth for evaluation of the tracking algorithm. A subset of 50 frames (about 3.5 min) and size 581x562 pixel (650x630 um) was manually annotated in the phase shift minimum projections of a time-lapse recording of neutrophils (stimulation 100 ng/ml IL-8). For results of benchmarking our algorithm see S4 Fig. (ZIP) [file pone.0270456.s007.zip › 000111.bmp]

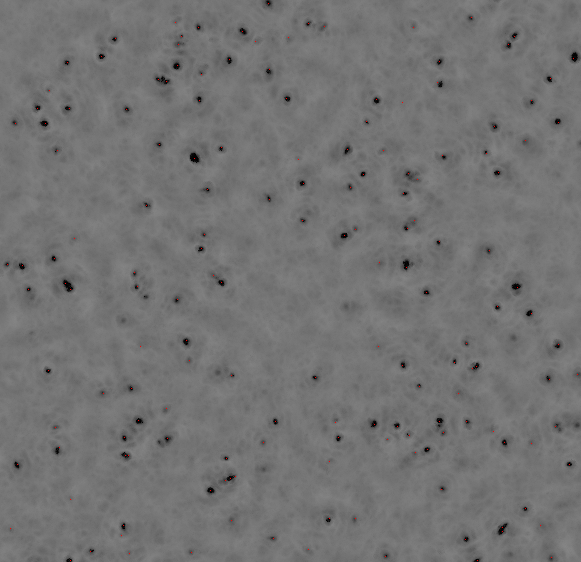

Supplement: S1 Dataset — Ground truth for evaluation of the tracking algorithm. A subset of 50 frames (about 3.5 min) and size 581x562 pixel (650x630 um) was manually annotated in the phase shift minimum projections of a time-lapse recording of neutrophils (stimulation 100 ng/ml IL-8). For results of benchmarking our algorithm see S4 Fig. (ZIP) [file pone.0270456.s007.zip › 000112.bmp]

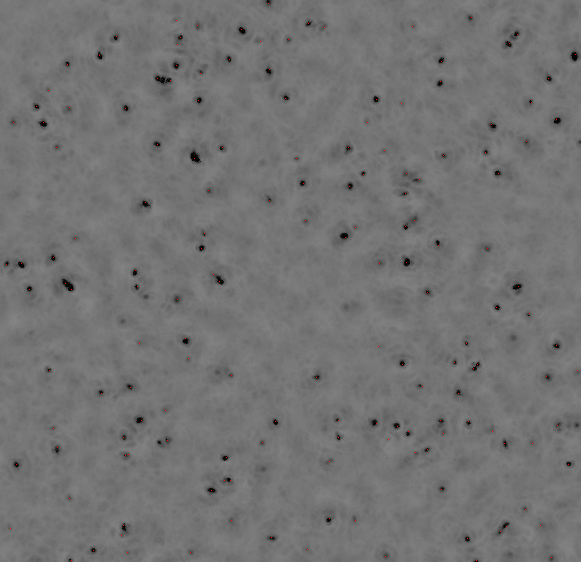

Supplement: S1 Dataset — Ground truth for evaluation of the tracking algorithm. A subset of 50 frames (about 3.5 min) and size 581x562 pixel (650x630 um) was manually annotated in the phase shift minimum projections of a time-lapse recording of neutrophils (stimulation 100 ng/ml IL-8). For results of benchmarking our algorithm see S4 Fig. (ZIP) [file pone.0270456.s007.zip › 000113.bmp]

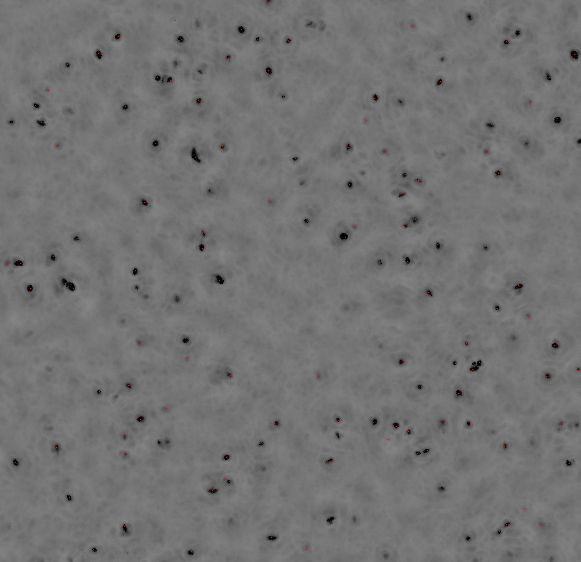

Supplement: S1 Dataset — Ground truth for evaluation of the tracking algorithm. A subset of 50 frames (about 3.5 min) and size 581x562 pixel (650x630 um) was manually annotated in the phase shift minimum projections of a time-lapse recording of neutrophils (stimulation 100 ng/ml IL-8). For results of benchmarking our algorithm see S4 Fig. (ZIP) [file pone.0270456.s007.zip › 000114.bmp]

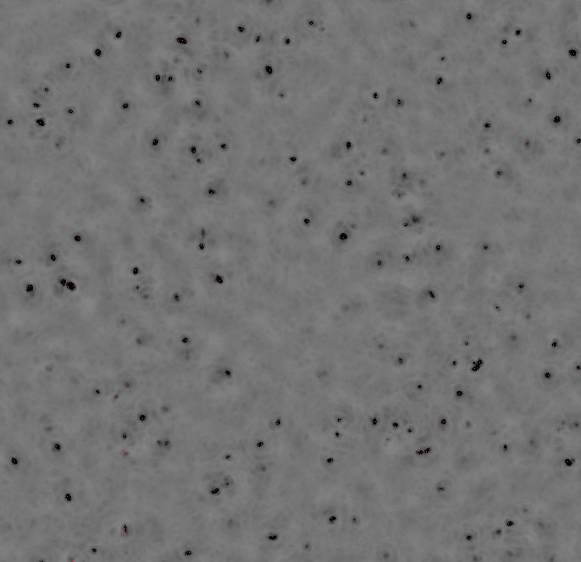

Supplement: S1 Dataset — Ground truth for evaluation of the tracking algorithm. A subset of 50 frames (about 3.5 min) and size 581x562 pixel (650x630 um) was manually annotated in the phase shift minimum projections of a time-lapse recording of neutrophils (stimulation 100 ng/ml IL-8). For results of benchmarking our algorithm see S4 Fig. (ZIP) [file pone.0270456.s007.zip › 000115.bmp]

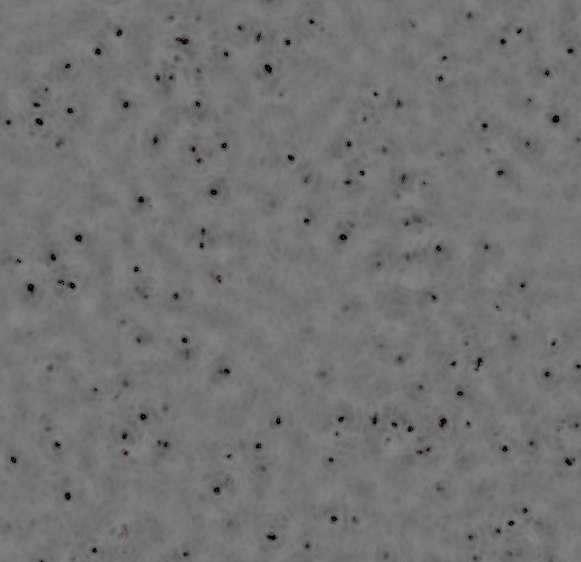

Supplement: S1 Dataset — Ground truth for evaluation of the tracking algorithm. A subset of 50 frames (about 3.5 min) and size 581x562 pixel (650x630 um) was manually annotated in the phase shift minimum projections of a time-lapse recording of neutrophils (stimulation 100 ng/ml IL-8). For results of benchmarking our algorithm see S4 Fig. (ZIP) [file pone.0270456.s007.zip › 000116.bmp]

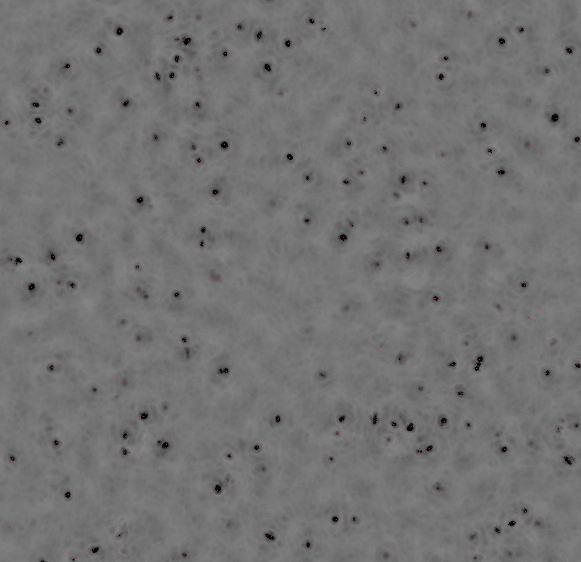

Supplement: S1 Dataset — Ground truth for evaluation of the tracking algorithm. A subset of 50 frames (about 3.5 min) and size 581x562 pixel (650x630 um) was manually annotated in the phase shift minimum projections of a time-lapse recording of neutrophils (stimulation 100 ng/ml IL-8). For results of benchmarking our algorithm see S4 Fig. (ZIP) [file pone.0270456.s007.zip › 000117.bmp]

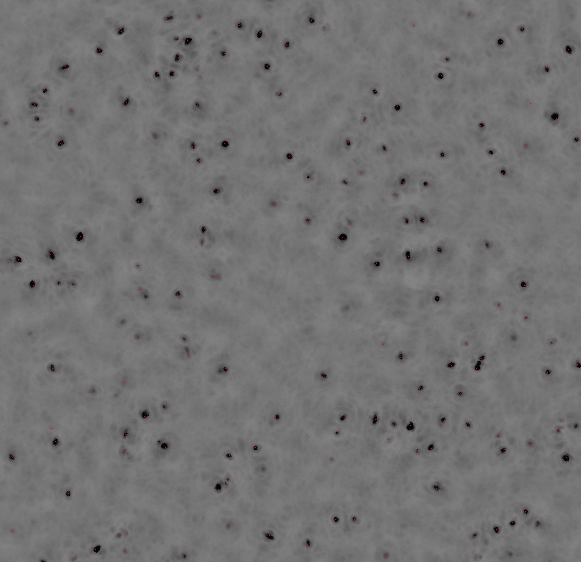

Supplement: S1 Dataset — Ground truth for evaluation of the tracking algorithm. A subset of 50 frames (about 3.5 min) and size 581x562 pixel (650x630 um) was manually annotated in the phase shift minimum projections of a time-lapse recording of neutrophils (stimulation 100 ng/ml IL-8). For results of benchmarking our algorithm see S4 Fig. (ZIP) [file pone.0270456.s007.zip › 000118.bmp]

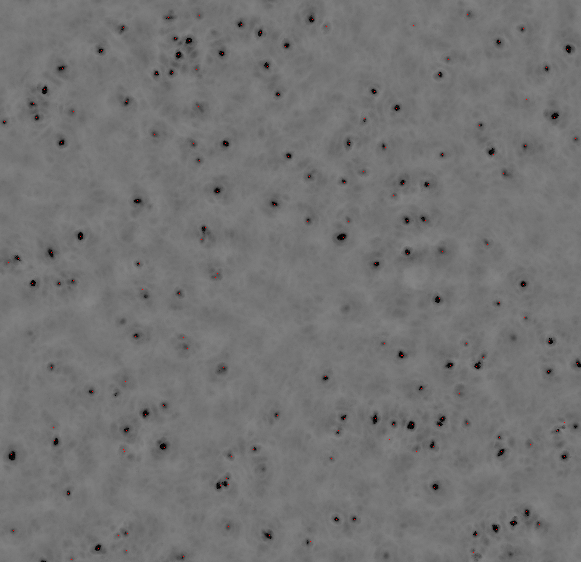

Supplement: S1 Dataset — Ground truth for evaluation of the tracking algorithm. A subset of 50 frames (about 3.5 min) and size 581x562 pixel (650x630 um) was manually annotated in the phase shift minimum projections of a time-lapse recording of neutrophils (stimulation 100 ng/ml IL-8). For results of benchmarking our algorithm see S4 Fig. (ZIP) [file pone.0270456.s007.zip › 000119.bmp]

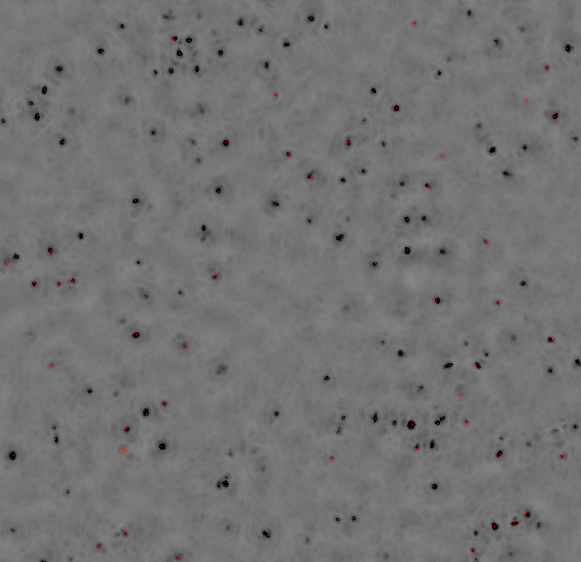

Supplement: S1 Dataset — Ground truth for evaluation of the tracking algorithm. A subset of 50 frames (about 3.5 min) and size 581x562 pixel (650x630 um) was manually annotated in the phase shift minimum projections of a time-lapse recording of neutrophils (stimulation 100 ng/ml IL-8). For results of benchmarking our algorithm see S4 Fig. (ZIP) [file pone.0270456.s007.zip › 000120.bmp]

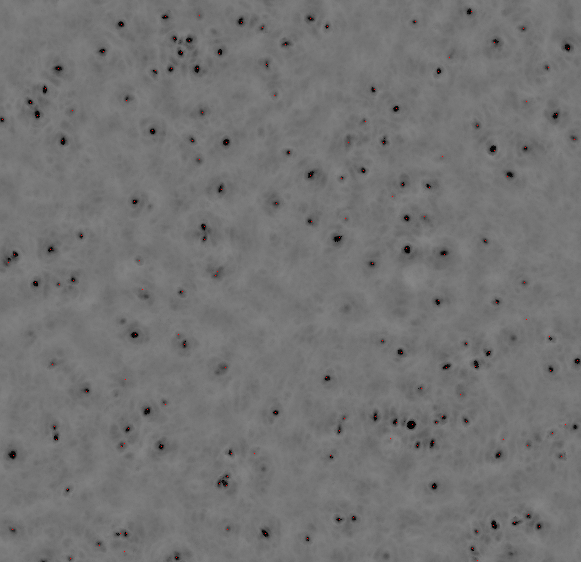

Supplement: S1 Dataset — Ground truth for evaluation of the tracking algorithm. A subset of 50 frames (about 3.5 min) and size 581x562 pixel (650x630 um) was manually annotated in the phase shift minimum projections of a time-lapse recording of neutrophils (stimulation 100 ng/ml IL-8). For results of benchmarking our algorithm see S4 Fig. (ZIP) [file pone.0270456.s007.zip › 000121.bmp]

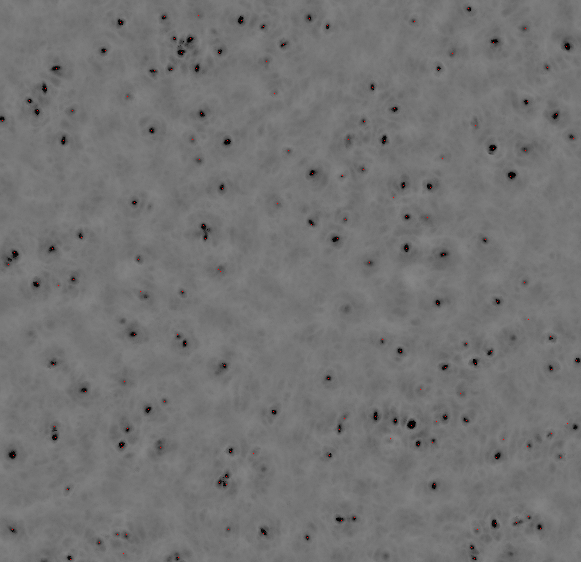

Supplement: S1 Dataset — Ground truth for evaluation of the tracking algorithm. A subset of 50 frames (about 3.5 min) and size 581x562 pixel (650x630 um) was manually annotated in the phase shift minimum projections of a time-lapse recording of neutrophils (stimulation 100 ng/ml IL-8). For results of benchmarking our algorithm see S4 Fig. (ZIP) [file pone.0270456.s007.zip › 000122.bmp]

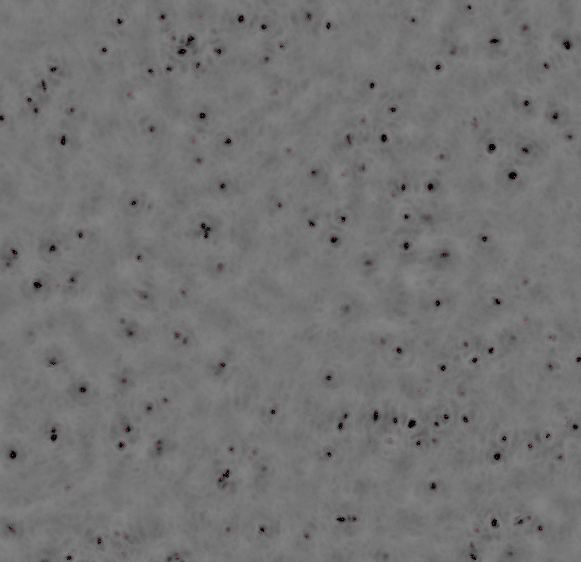

Supplement: S1 Dataset — Ground truth for evaluation of the tracking algorithm. A subset of 50 frames (about 3.5 min) and size 581x562 pixel (650x630 um) was manually annotated in the phase shift minimum projections of a time-lapse recording of neutrophils (stimulation 100 ng/ml IL-8). For results of benchmarking our algorithm see S4 Fig. (ZIP) [file pone.0270456.s007.zip › 000123.bmp]

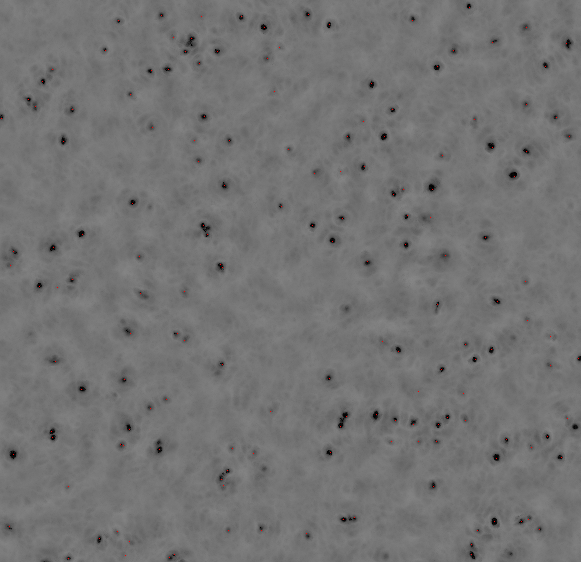

Supplement: S1 Dataset — Ground truth for evaluation of the tracking algorithm. A subset of 50 frames (about 3.5 min) and size 581x562 pixel (650x630 um) was manually annotated in the phase shift minimum projections of a time-lapse recording of neutrophils (stimulation 100 ng/ml IL-8). For results of benchmarking our algorithm see S4 Fig. (ZIP) [file pone.0270456.s007.zip › 000124.bmp]

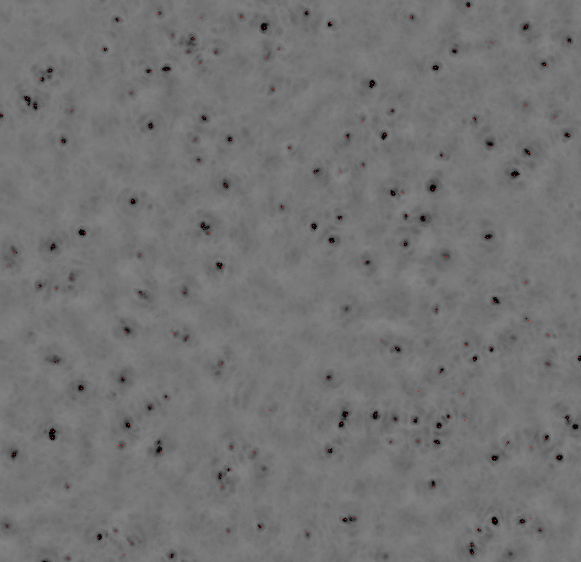

Supplement: S1 Dataset — Ground truth for evaluation of the tracking algorithm. A subset of 50 frames (about 3.5 min) and size 581x562 pixel (650x630 um) was manually annotated in the phase shift minimum projections of a time-lapse recording of neutrophils (stimulation 100 ng/ml IL-8). For results of benchmarking our algorithm see S4 Fig. (ZIP) [file pone.0270456.s007.zip › 000125.bmp]

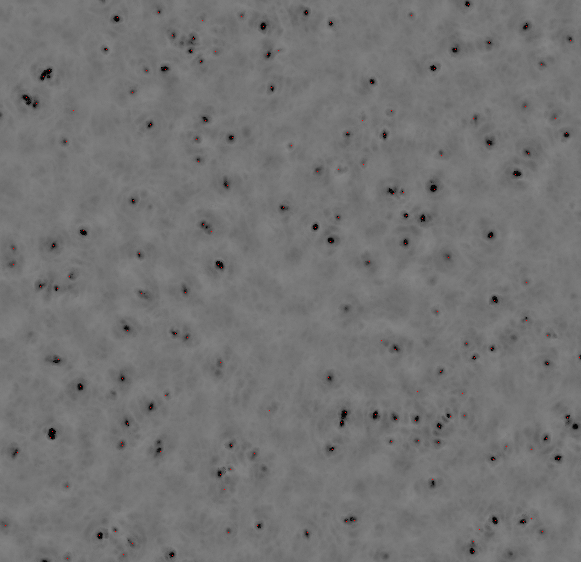

Supplement: S1 Dataset — Ground truth for evaluation of the tracking algorithm. A subset of 50 frames (about 3.5 min) and size 581x562 pixel (650x630 um) was manually annotated in the phase shift minimum projections of a time-lapse recording of neutrophils (stimulation 100 ng/ml IL-8). For results of benchmarking our algorithm see S4 Fig. (ZIP) [file pone.0270456.s007.zip › 000126.bmp]

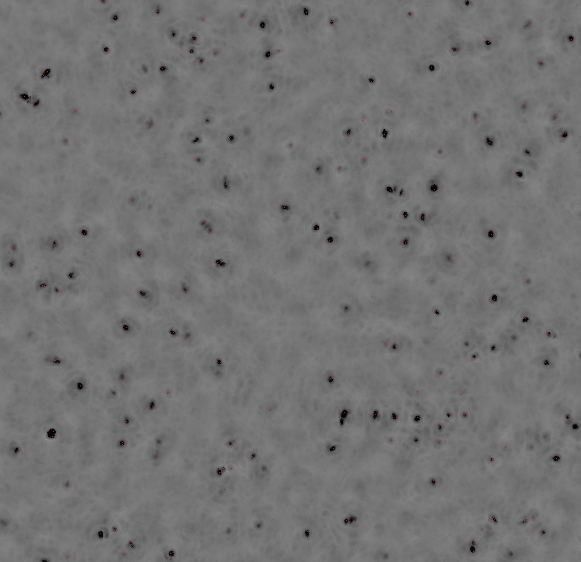

Supplement: S1 Dataset — Ground truth for evaluation of the tracking algorithm. A subset of 50 frames (about 3.5 min) and size 581x562 pixel (650x630 um) was manually annotated in the phase shift minimum projections of a time-lapse recording of neutrophils (stimulation 100 ng/ml IL-8). For results of benchmarking our algorithm see S4 Fig. (ZIP) [file pone.0270456.s007.zip › 000127.bmp]

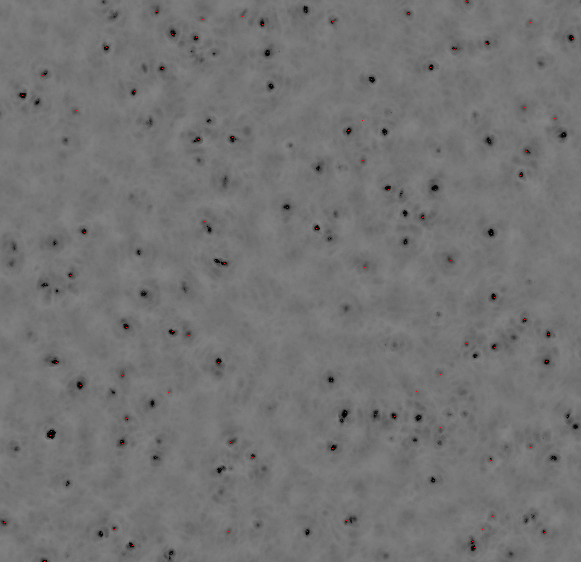

Supplement: S1 Dataset — Ground truth for evaluation of the tracking algorithm. A subset of 50 frames (about 3.5 min) and size 581x562 pixel (650x630 um) was manually annotated in the phase shift minimum projections of a time-lapse recording of neutrophils (stimulation 100 ng/ml IL-8). For results of benchmarking our algorithm see S4 Fig. (ZIP) [file pone.0270456.s007.zip › 000128.bmp]

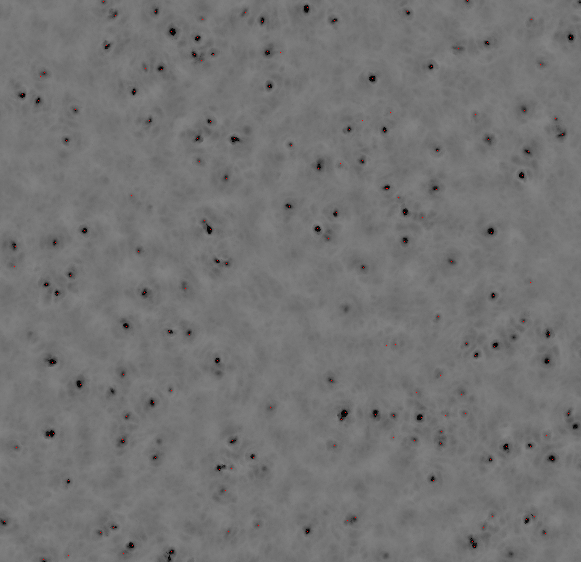

Supplement: S1 Dataset — Ground truth for evaluation of the tracking algorithm. A subset of 50 frames (about 3.5 min) and size 581x562 pixel (650x630 um) was manually annotated in the phase shift minimum projections of a time-lapse recording of neutrophils (stimulation 100 ng/ml IL-8). For results of benchmarking our algorithm see S4 Fig. (ZIP) [file pone.0270456.s007.zip › 000129.bmp]

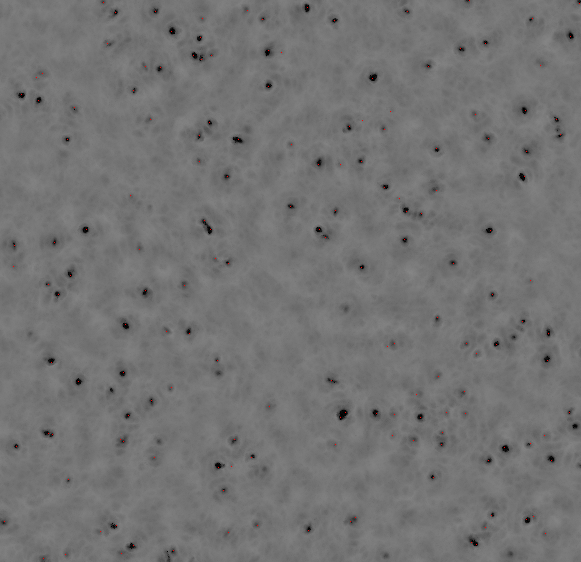

Supplement: S1 Dataset — Ground truth for evaluation of the tracking algorithm. A subset of 50 frames (about 3.5 min) and size 581x562 pixel (650x630 um) was manually annotated in the phase shift minimum projections of a time-lapse recording of neutrophils (stimulation 100 ng/ml IL-8). For results of benchmarking our algorithm see S4 Fig. (ZIP) [file pone.0270456.s007.zip › 000130.bmp]

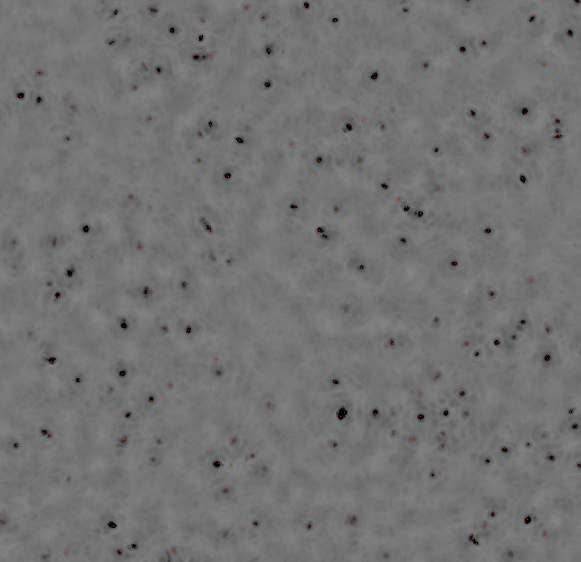

Supplement: S1 Dataset — Ground truth for evaluation of the tracking algorithm. A subset of 50 frames (about 3.5 min) and size 581x562 pixel (650x630 um) was manually annotated in the phase shift minimum projections of a time-lapse recording of neutrophils (stimulation 100 ng/ml IL-8). For results of benchmarking our algorithm see S4 Fig. (ZIP) [file pone.0270456.s007.zip › 000131.bmp]

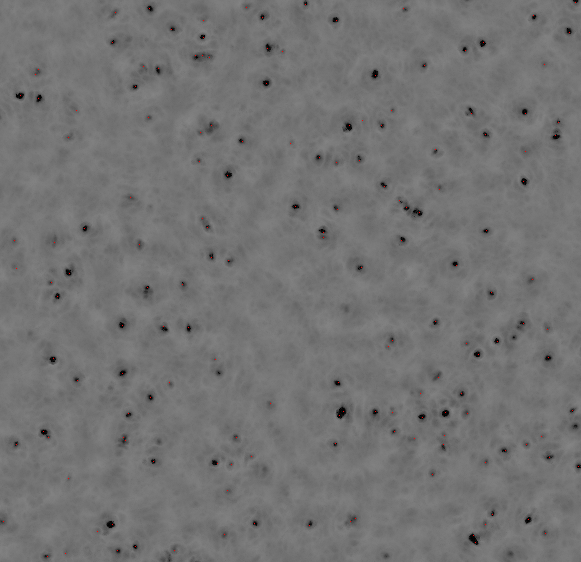

Supplement: S1 Dataset — Ground truth for evaluation of the tracking algorithm. A subset of 50 frames (about 3.5 min) and size 581x562 pixel (650x630 um) was manually annotated in the phase shift minimum projections of a time-lapse recording of neutrophils (stimulation 100 ng/ml IL-8). For results of benchmarking our algorithm see S4 Fig. (ZIP) [file pone.0270456.s007.zip › 000132.bmp]

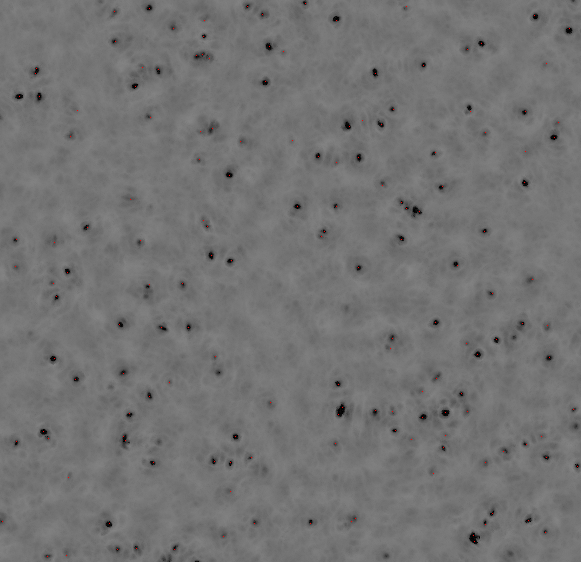

Supplement: S1 Dataset — Ground truth for evaluation of the tracking algorithm. A subset of 50 frames (about 3.5 min) and size 581x562 pixel (650x630 um) was manually annotated in the phase shift minimum projections of a time-lapse recording of neutrophils (stimulation 100 ng/ml IL-8). For results of benchmarking our algorithm see S4 Fig. (ZIP) [file pone.0270456.s007.zip › 000133.bmp]

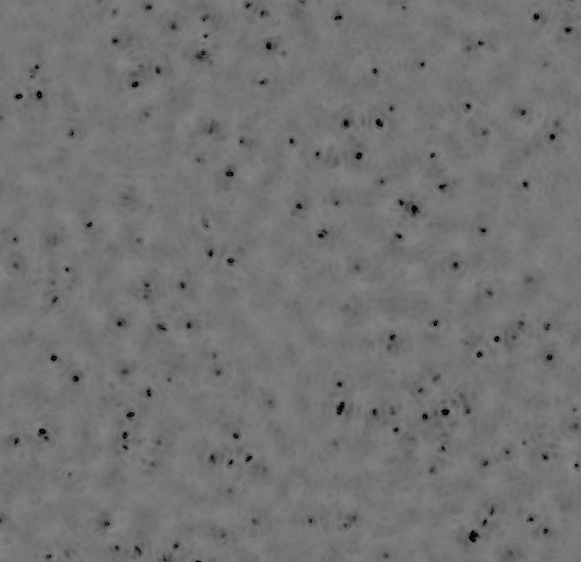

Supplement: S1 Dataset — Ground truth for evaluation of the tracking algorithm. A subset of 50 frames (about 3.5 min) and size 581x562 pixel (650x630 um) was manually annotated in the phase shift minimum projections of a time-lapse recording of neutrophils (stimulation 100 ng/ml IL-8). For results of benchmarking our algorithm see S4 Fig. (ZIP) [file pone.0270456.s007.zip › 000134.bmp]

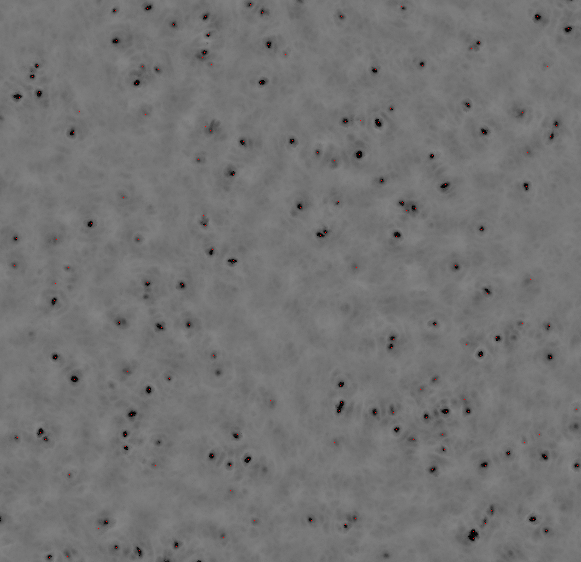

Supplement: S1 Dataset — Ground truth for evaluation of the tracking algorithm. A subset of 50 frames (about 3.5 min) and size 581x562 pixel (650x630 um) was manually annotated in the phase shift minimum projections of a time-lapse recording of neutrophils (stimulation 100 ng/ml IL-8). For results of benchmarking our algorithm see S4 Fig. (ZIP) [file pone.0270456.s007.zip › 000135.bmp]

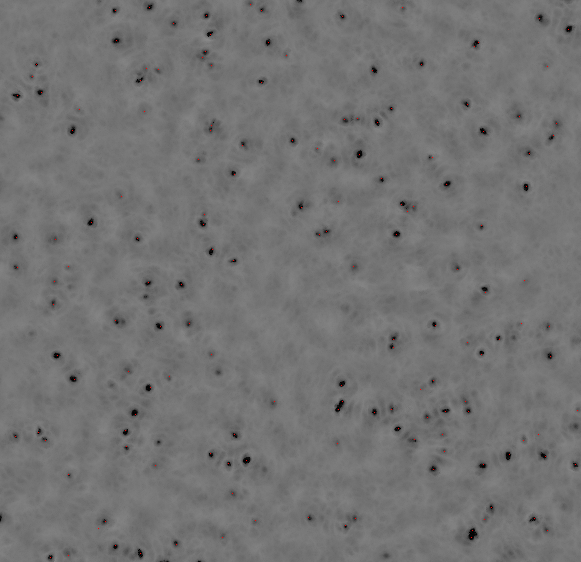

Supplement: S1 Dataset — Ground truth for evaluation of the tracking algorithm. A subset of 50 frames (about 3.5 min) and size 581x562 pixel (650x630 um) was manually annotated in the phase shift minimum projections of a time-lapse recording of neutrophils (stimulation 100 ng/ml IL-8). For results of benchmarking our algorithm see S4 Fig. (ZIP) [file pone.0270456.s007.zip › 000136.bmp]

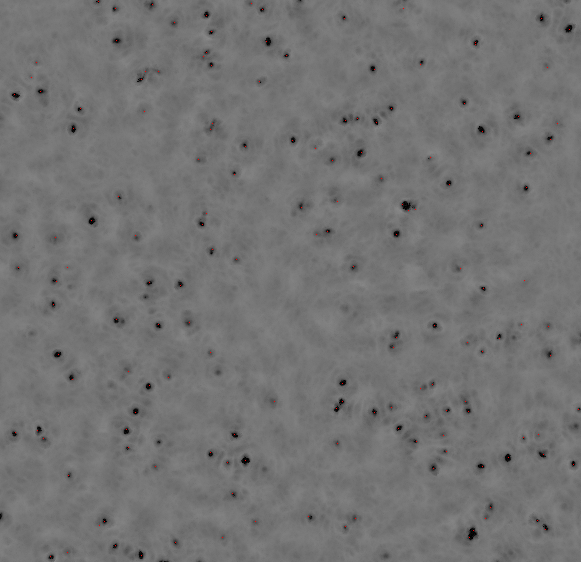

Supplement: S1 Dataset — Ground truth for evaluation of the tracking algorithm. A subset of 50 frames (about 3.5 min) and size 581x562 pixel (650x630 um) was manually annotated in the phase shift minimum projections of a time-lapse recording of neutrophils (stimulation 100 ng/ml IL-8). For results of benchmarking our algorithm see S4 Fig. (ZIP) [file pone.0270456.s007.zip › 000137.bmp]

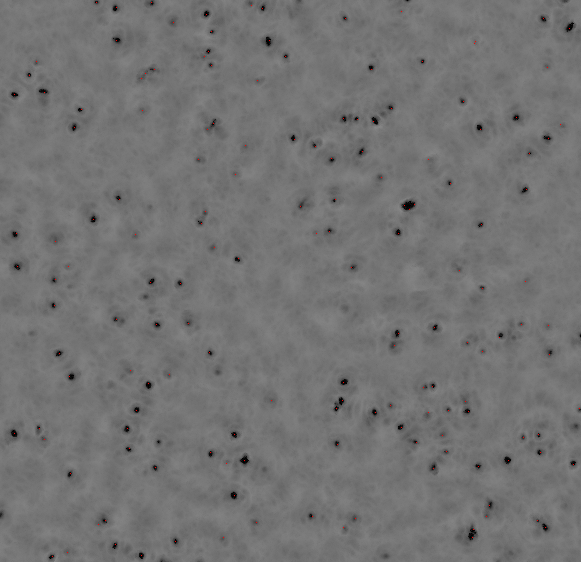

Supplement: S1 Dataset — Ground truth for evaluation of the tracking algorithm. A subset of 50 frames (about 3.5 min) and size 581x562 pixel (650x630 um) was manually annotated in the phase shift minimum projections of a time-lapse recording of neutrophils (stimulation 100 ng/ml IL-8). For results of benchmarking our algorithm see S4 Fig. (ZIP) [file pone.0270456.s007.zip › 000138.bmp]

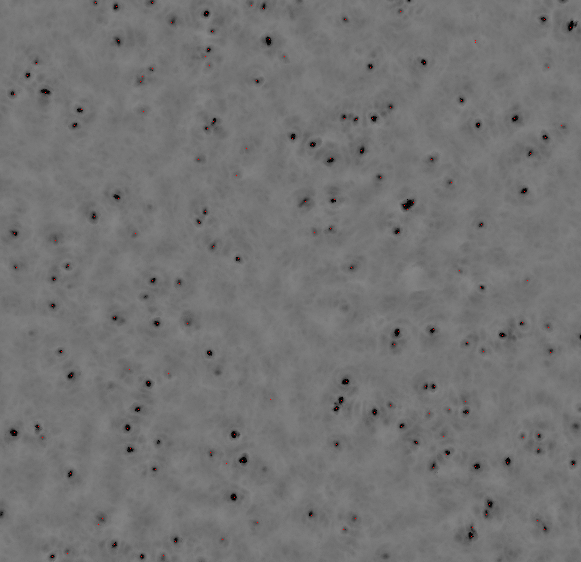

Supplement: S1 Dataset — Ground truth for evaluation of the tracking algorithm. A subset of 50 frames (about 3.5 min) and size 581x562 pixel (650x630 um) was manually annotated in the phase shift minimum projections of a time-lapse recording of neutrophils (stimulation 100 ng/ml IL-8). For results of benchmarking our algorithm see S4 Fig. (ZIP) [file pone.0270456.s007.zip › 000139.bmp]

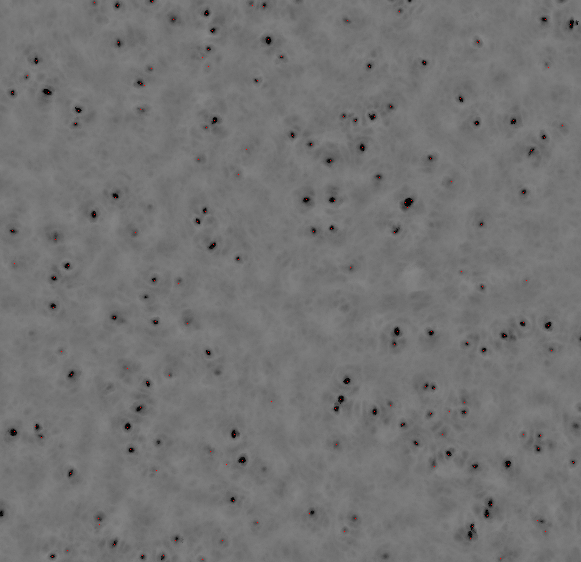

Supplement: S1 Dataset — Ground truth for evaluation of the tracking algorithm. A subset of 50 frames (about 3.5 min) and size 581x562 pixel (650x630 um) was manually annotated in the phase shift minimum projections of a time-lapse recording of neutrophils (stimulation 100 ng/ml IL-8). For results of benchmarking our algorithm see S4 Fig. (ZIP) [file pone.0270456.s007.zip › 000140.bmp]

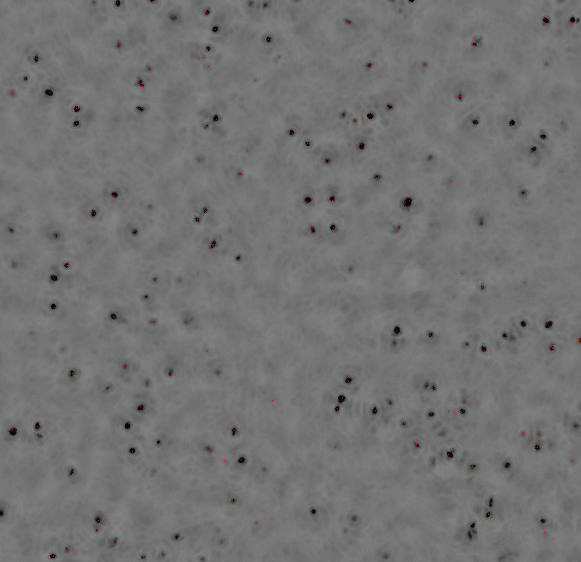

Supplement: S1 Dataset — Ground truth for evaluation of the tracking algorithm. A subset of 50 frames (about 3.5 min) and size 581x562 pixel (650x630 um) was manually annotated in the phase shift minimum projections of a time-lapse recording of neutrophils (stimulation 100 ng/ml IL-8). For results of benchmarking our algorithm see S4 Fig. (ZIP) [file pone.0270456.s007.zip › 000141.bmp]

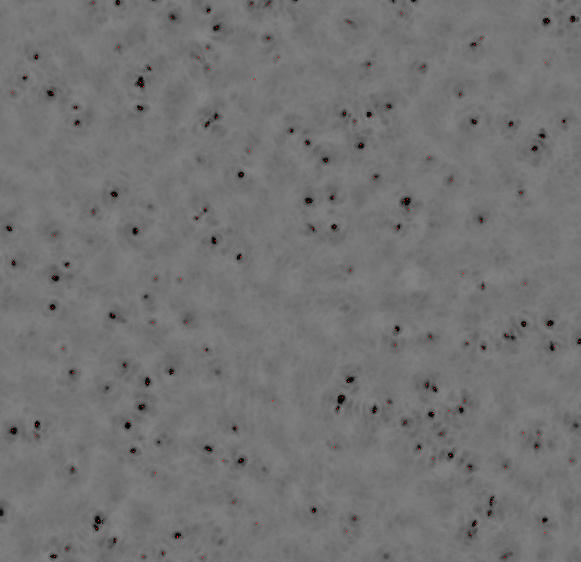

Supplement: S1 Dataset — Ground truth for evaluation of the tracking algorithm. A subset of 50 frames (about 3.5 min) and size 581x562 pixel (650x630 um) was manually annotated in the phase shift minimum projections of a time-lapse recording of neutrophils (stimulation 100 ng/ml IL-8). For results of benchmarking our algorithm see S4 Fig. (ZIP) [file pone.0270456.s007.zip › 000142.bmp]

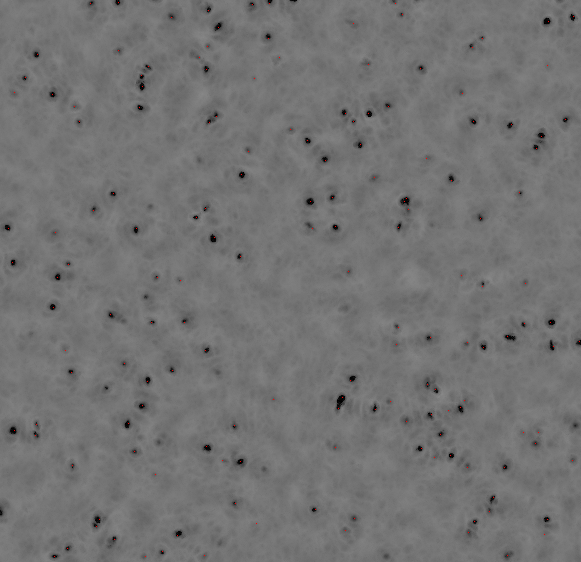

Supplement: S1 Dataset — Ground truth for evaluation of the tracking algorithm. A subset of 50 frames (about 3.5 min) and size 581x562 pixel (650x630 um) was manually annotated in the phase shift minimum projections of a time-lapse recording of neutrophils (stimulation 100 ng/ml IL-8). For results of benchmarking our algorithm see S4 Fig. (ZIP) [file pone.0270456.s007.zip › 000143.bmp]

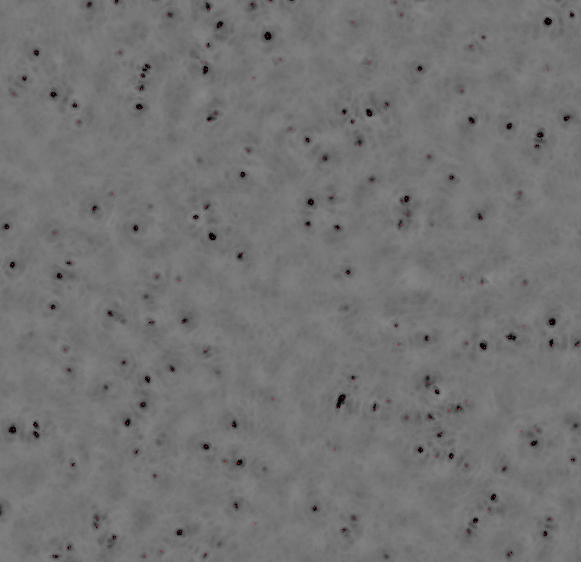

Supplement: S1 Dataset — Ground truth for evaluation of the tracking algorithm. A subset of 50 frames (about 3.5 min) and size 581x562 pixel (650x630 um) was manually annotated in the phase shift minimum projections of a time-lapse recording of neutrophils (stimulation 100 ng/ml IL-8). For results of benchmarking our algorithm see S4 Fig. (ZIP) [file pone.0270456.s007.zip › 000144.bmp]

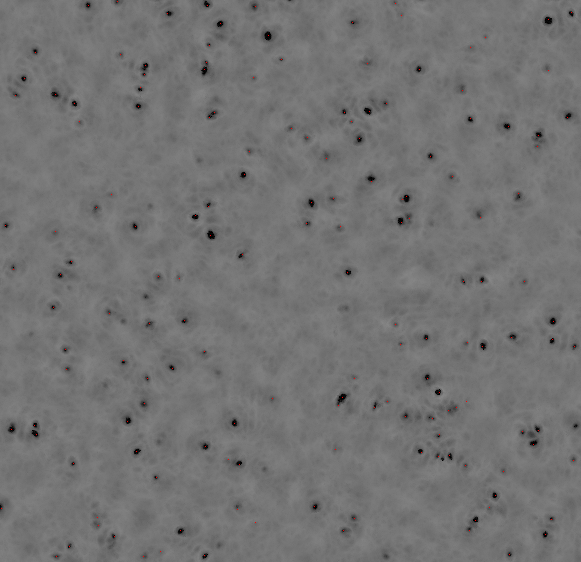

Supplement: S1 Dataset — Ground truth for evaluation of the tracking algorithm. A subset of 50 frames (about 3.5 min) and size 581x562 pixel (650x630 um) was manually annotated in the phase shift minimum projections of a time-lapse recording of neutrophils (stimulation 100 ng/ml IL-8). For results of benchmarking our algorithm see S4 Fig. (ZIP) [file pone.0270456.s007.zip › 000145.bmp]

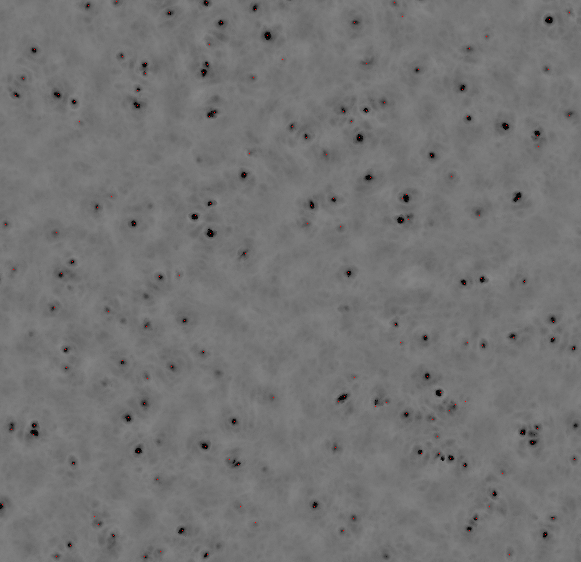

Supplement: S1 Dataset — Ground truth for evaluation of the tracking algorithm. A subset of 50 frames (about 3.5 min) and size 581x562 pixel (650x630 um) was manually annotated in the phase shift minimum projections of a time-lapse recording of neutrophils (stimulation 100 ng/ml IL-8). For results of benchmarking our algorithm see S4 Fig. (ZIP) [file pone.0270456.s007.zip › 000146.bmp]

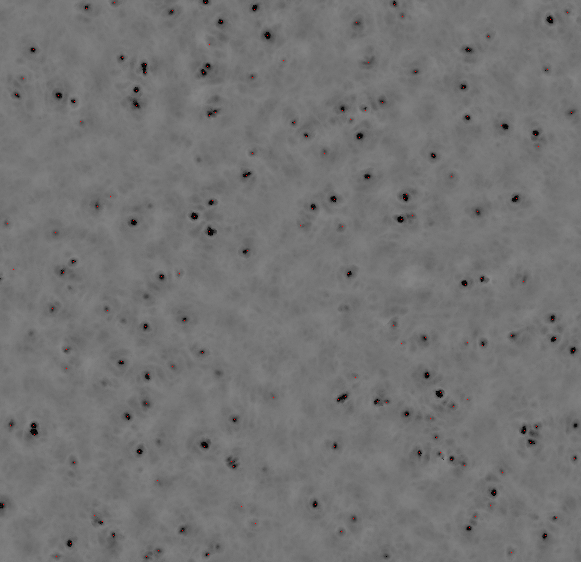

Supplement: S1 Dataset — Ground truth for evaluation of the tracking algorithm. A subset of 50 frames (about 3.5 min) and size 581x562 pixel (650x630 um) was manually annotated in the phase shift minimum projections of a time-lapse recording of neutrophils (stimulation 100 ng/ml IL-8). For results of benchmarking our algorithm see S4 Fig. (ZIP) [file pone.0270456.s007.zip › 000147.bmp]

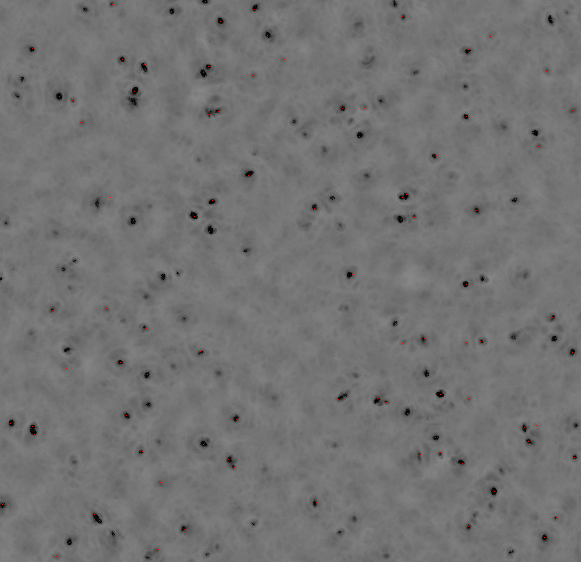

Supplement: S1 Dataset — Ground truth for evaluation of the tracking algorithm. A subset of 50 frames (about 3.5 min) and size 581x562 pixel (650x630 um) was manually annotated in the phase shift minimum projections of a time-lapse recording of neutrophils (stimulation 100 ng/ml IL-8). For results of benchmarking our algorithm see S4 Fig. (ZIP) [file pone.0270456.s007.zip › 000148.bmp]

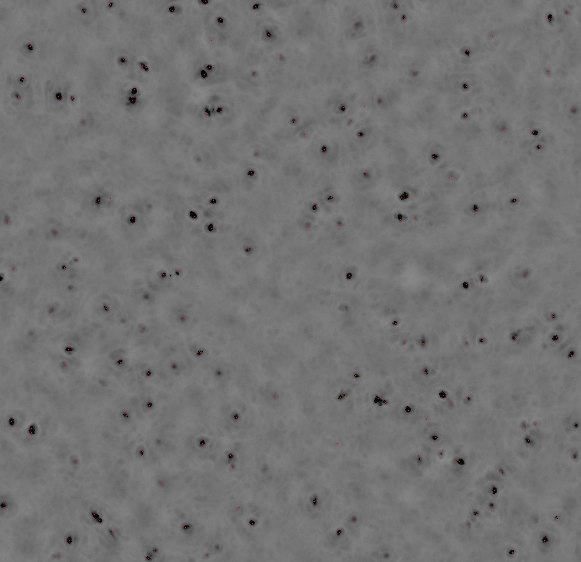

Supplement: S1 Dataset — Ground truth for evaluation of the tracking algorithm. A subset of 50 frames (about 3.5 min) and size 581x562 pixel (650x630 um) was manually annotated in the phase shift minimum projections of a time-lapse recording of neutrophils (stimulation 100 ng/ml IL-8). For results of benchmarking our algorithm see S4 Fig. (ZIP) [file pone.0270456.s007.zip › 000149.bmp]

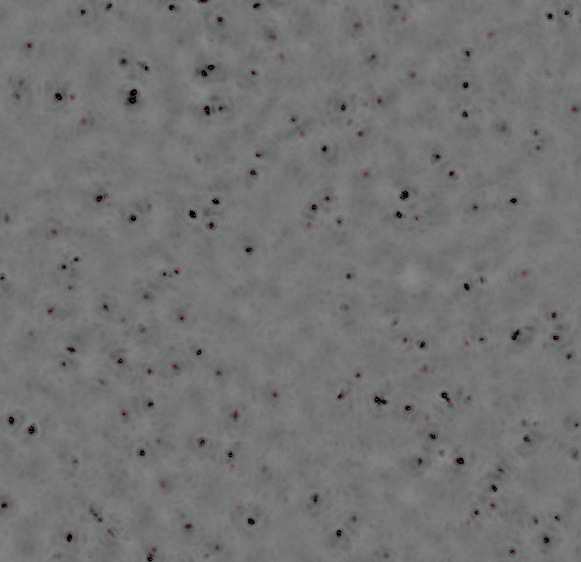

Supplement: S1 Dataset — Ground truth for evaluation of the tracking algorithm. A subset of 50 frames (about 3.5 min) and size 581x562 pixel (650x630 um) was manually annotated in the phase shift minimum projections of a time-lapse recording of neutrophils (stimulation 100 ng/ml IL-8). For results of benchmarking our algorithm see S4 Fig. (ZIP) [file pone.0270456.s007.zip › 000150.bmp]
