# Supplementary material for: Method for simultaneous tracking of thousands of unlabeled cells within a transparent 3D matrix
Source: PLoS One. 2022 Jun 24;17(6):e0270456. doi: 10.1371/journal.pone.0270456 (PMC9232129; doi:10.1371/journal.pone.0270456)
Supplement: S5 Fig — We compared the migration velocity towards the gradient of IL-8- and fMLP-stimulated neutrophils with their respective control groups (n = 3 in every of the four groups). To do so, we averaged over 75 values of the outward migration velocities starting after 2.5 min (i.e. 5 minutes of tracking, leaving out the onset of migration, cf. Fig 7A and 7B). The result of these comparisons is displayed for IL-8 (left) and fMLP (right). It can be seen that IL-8 might be better suited than fMLP for telling apart asthma from non-asthma-conditions (p-values of 0.03* and 0.09 for IL-8 and fMLP, respectively). (PDF) [file pone.0270456.s005.pdf]

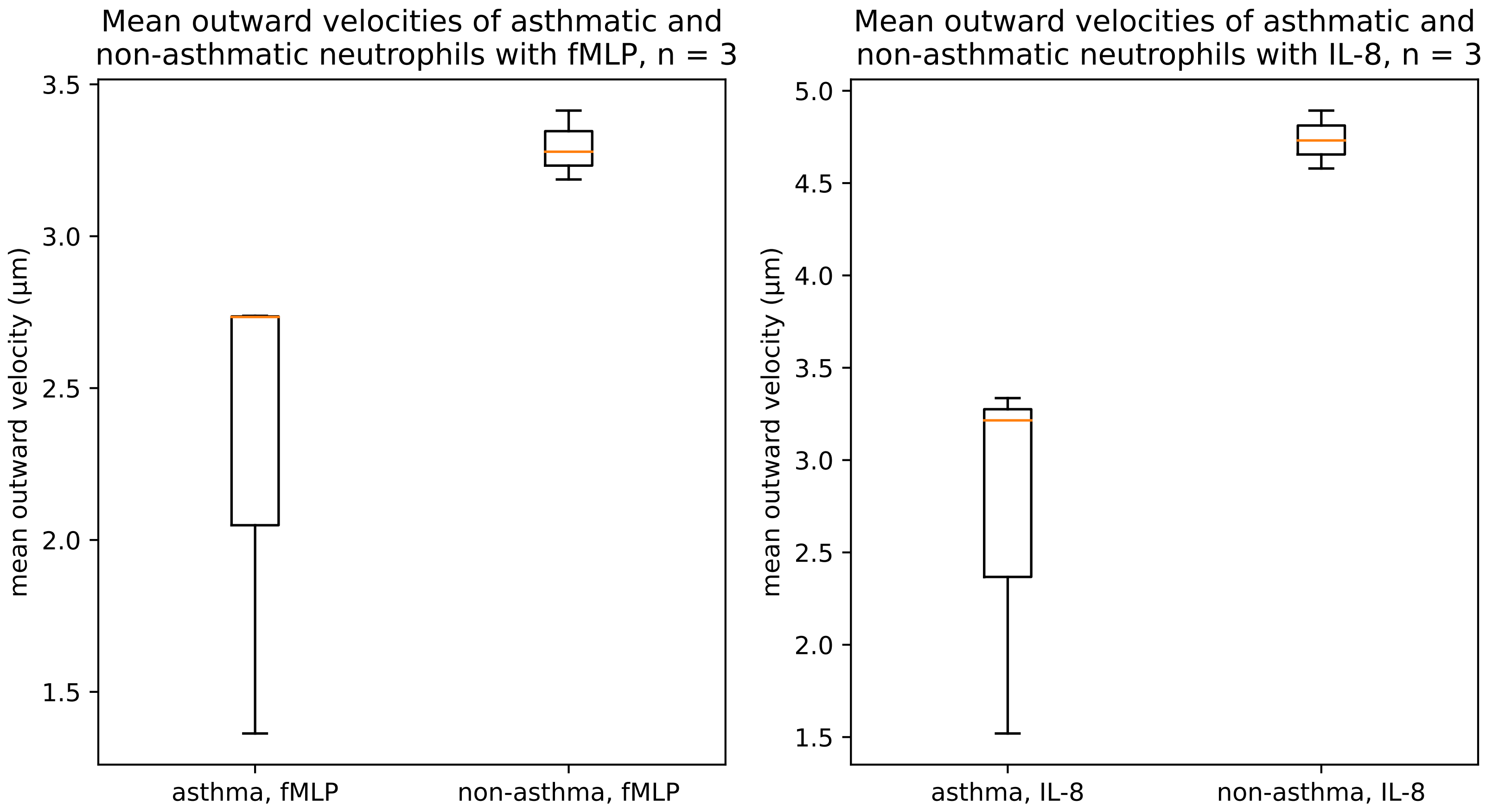
Mean outward velocities of asthmatic and non-asthmatic neutrophils with fMLP, n = 3
mean outward velocity (µm)
3.5
3.0
2.5
2.0
1.5
asthma, fMLP
non-asthma, fMLP
Mean outward velocities of asthmatic and non-asthmatic neutrophils with IL-8, n = 3
mean outward velocity (µm)
5.0
4.5
4.0
3.5
3.0
2.5
2.0
1.5
asthma, IL-8
non-asthma, IL-8
