# Supplementary material for: Method for simultaneous tracking of thousands of unlabeled cells within a transparent 3D matrix
Source: PLoS One. 2022 Jun 24;17(6):e0270456. doi: 10.1371/journal.pone.0270456 (PMC9232129; doi:10.1371/journal.pone.0270456)
Supplement: Attachment — Submitted filename: Response_to_Reviewers.docx [file pone.0270456.s008.docx]

**Response to the reviewers**

Reviewer #1

*1. The evaluation of the measurements of cell detection, cell position and cell tracking is not well described, it is not supported by proper measurements and proper calculations. I strongly encourage the author to improve these assessments to better characterize the methodology.*

*Cell detection should be evaluated in terms of precision and recall calculated from comparison measurements made with a reference measurement, i.e., another reference microscope, such as fluorescence microscopy.*

*Similarly, cell position should be assessed with precision measurements obtained by comparing lens-free microscopy to another reference microscopy. Here, the authors simply state "we estimate that the accuracy that can be obtained from these reconstructions is 30 µm in Z position and 1.5 µm in X and Y position." This is not a robust assessment. As an example, please follow the methodology discussed in the supplementary information of 'Berdeu et al.'*

*Finally the cell tracking efficiency should be assessed with a given tracking index calculated with a series of annotated ground truth cell tracking. The methodology is well described in 'Ulman, V., Maška, M., Magnusson, K. E., Ronneberger, O., Haubold, C., Harder, N., ... & Ortiz-de-Solorzano, C. (2017).*

*An objective comparison of cell-tracking algorithms. Nature methods, 14(12), 1141-1152.'. I disagree with the authors when they write ‘Tracking reliability can be assessed quantitatively by analyzing the distribution of track lengths.’.*

*In sum, from my point of view further assessments are needed, they are very important to characterize the overall methodology discussed in this paper.*

The Reviewer’s first point raises the question of determining the precision of our holographic setup for localizing cells. A real reference measurement would require to spatially arrange cells in known places to serve as a 3D-calibration sample, which, unfortunately, we found no way of doing. Therefore, we have made comparative measurements using two types of synthetic micro beads, as described in the manuscript (page 13, paragraph 1) and in more detail below (the respective passages in the manuscript pp 13ff have been revised accordingly):

To determine the precision of our setup, Reviewer 1 proposes to recreate the measurements of Berdeu et al. We have tried so, using Polydimethysiloxan (PDMS) as a 3D-Matrix (collagen proved too soft to keep the microbeads in position when transferring the sample from one microscope to the other). However, it turned out to be difficult to localize the microbeads in PDMS using our holographic setup because the phase as well as the amplitude contrast were too weak to be reliably detected. We have therefore employed two different methods to measure the accuracy in x-y and in z, respectively: (1) X-Y-accuracy was determined using a conventional calibration target (Carl Zeiss Microscope Stage Micrometer; see Fig A in S4 Fig). The total length of the calibration scale is 1 mm (+- 1 µm) and was determined to be 1042 µm with our holographic setup (measured in the amplitude representation of the holographic reconstruction), corresponding to a lateral precision of 4.2 %. (2) Accuracy in z-position was determined as proposed by the reviewer similar to Berdeu et al. using fluorescent microbeads (BD Biosciences “Calibrite” unlabeled, PMMA, diameter 6 µm). The measurements were carried out in the same micro-dish as our other measurements (ibidi µ-dish), but with a glass cover slip put into the dish to partially cover the bottom. After the microbeads-suspension was added, some of the beads came to lay on top of the cover slip, while others sank on the bottom of the dish. We have determined the z-positions of both classes of beads with a laser scanning microscope (Zeiss LSM 510) and with our holographic setup and compared the z-positions determined with these two methods. (The exact z-positions in the holographic data have been determined similar to Berdeu et al. through finding the maxima of filtering the reconstructed 3D-data with a reference bead-pattern and taking the highest intensity of the x-z-cross sections in the fluorescent LSM images, see S4 Fig.). The results of this comparison can be seen in Fig C in S4 Fig; the z-positions found with our holographic setup have a slightly higher standard deviation than the those of the reference, but the overall accuracy of our setup (with microbeads) is within ~6% of the reference microscope (LSM: 120.9 μm average z-distance between upper and lower beads / holographic setup: 127.9 μm). The manuscript has been revised to include these more precise additional measurements (cf. pp 13). As stated in the manuscript, the true accuracy of our setup in determining cell positions will be lower than that, because cells are much less defined objects with a greater variety of shapes, sizes and chemical composition (and therefore phase shifts) than microbeads.

Reviewer 1 also asks to determine the performance of the tracking algorithm using a benchmark data set. The algorithm itself (trackpy; https://zenodo.org/record/3492186) is a direct Python-implementation of the widely used tracking algorithm of Crocker and Grier, which was originally written in IDL (http://doi.org/10.1006/jcis.1996.0217). The original algorithm has been cited over 2600 times and has been used for particle tracking and cell tracking; an implemention by Willemse et al. has been benchmarked as well (see Chenouard, et al., Nat Methods 11, 281–289 (2014). https://doi.org/10.1038/nmeth.2808). The reviewer proposes to adopt the benchmarking scheme of Ulman et al. to measure the tracking efficiency of the algorithm in our setup. However, the benchmarking scheme devised within this paper is not applicable to our algorithm, mainly because we do not detect cells as contiguous regions, but as points in three dimensional space (the Crocker-Grier-algorithm is a particle tracking algorithm as opposed to a cell/shape tracking algorithm). Consequently, the two main evaluation criteria of Ulman et al. (segmentation accuracy SEG and tracking accuracy TRA) can not be calculated within our tracking approach. SEG refers to the average overlap between detected cell regions and and ground truth; and can therefore not be calculated. Also, the tracking accuracy TRA is not applicable: TRA was invented by Matula et al. (https://doi.org/10.1371/journal.pone.0144959) and relies on overlap of cell shapes between successive frames as well. The authors explicitly state that "such a simple test does not exist for the particle tracking problem, where particles are considered volumeless, making similar evaluation procedure computationally unfeasible.".

However, to answer the reviewers valid question about the performance of our particular use of the Crocker-Grier-Algorithm, we have manually constructed a ground truth from our data (containing a subset of about 8000 cells over 50 frames, see S6 Dataset). From that, we determined (1) the number of falsely positive detected cells; (2) the number of missed (falsely negative detected) cells and (3) the average distance between computed tracks and tracks of the ground truth. To determine (1) and (2), we have looked at the nearest neighbors between the ground truth and the tracking results and counted every cell in the ground truth, for which no neighbor in the tracking results was found within a distance of 11.2 µm as a false negative result. Conversely, a cell in the tracking results, within 11.2 µm of which no cell in the ground truth could be found, was counted as false positive. The cutoff radius of 11.2 µm is somewhat arbitrary and was chosen such, because it was also the search radius of our tracking. Moreover, it lies well below the average nearest neighbor between the cells of the ground truth (cf. S5 Fig). The exact numbers found are: Total cells in ground truth: 8105; total cells found by algorithm: 8046; Total false positives: 775 (9.6 %) ; Total false negatives: 484 (6.0 %) . Finally, the average distance between true and computed tracks was found by comparing the distance distributions of nearest neighbors within the ground truth to that between ground truth and tracked cell positions. S5 Fig shows this average distance to be 4.2 µm (compared to an average of 26.6 µm between neighbouring cells). Though ideally, the distance should be close to zero, it can be seen that at the low cell densities used in our experiments, the algorithm will in most cases find the right cell. For our purpose of finding momentary migration values (speed, velocity, angle), these accuracies suffice, as is reflected by the fact, that we could reproduce the known migratory features of neutrophil granulocytes.

Lastly, Reviewer 1 raises the point that the distribution of track lengths cannot be assumed to be a good measure for tracking reliability. While this is true in general, we have a special case, because the number of tracked objects remains the same throughout the experiment. This holds, because (I) the collagen drop fits completely on the image sensor permitting to image all cells in the system; (II) no cells are added or taken out of the collagen drop; and (III), the observation time is rather short (20 min), making for only negligible amounts of cell disintegration, budding of vesicles etc. Hence, if cell detection and tracking were carried out perfectly, the result would be a set of tracks, one for each cell in the collagen drop, with a length of *n* frames. Therefore, the presence of tracks shorter than *n* indicates tracking errors. A similar argument is made in the paper of Ulman et al. (2017), which the reviewer cited. Therein, the fraction of complete tracks and the average of the longest continuous tracklets (given in percent of the complete track) are also mentioned and calculated as quality parameters of a given tracking algorithm.

*2. I have a concern dealing with the temperature control of the cell culture. If the CMOS sensor is very close to the cell and if it is too hot or not stable in temperature, it can influence the cell culture condition and consequently the cell measurements. This point is discussed in the conclusion, but there is no mention of this point in the Material and methods part nor in the Results part. I would suggest the author to conduct a thermal assay and present proper results to better ensure that CMOS heating is not influencing the culture conditions over time.*

The reviewer has a valid concern about possible (unwanted) heating of the cells through their proximity to the CMOS sensor. We have addressed this concern in our manuscript [p. 22/line 546ff]; in short, we have (1) measured the temperature of both the CMOS sensor and the medium-filled cell culture dish using a FLIR P660 infrared camera after one hour of continuous tracking and (2) made sure that a large enough volume of cell culture medium was used (1 ml) to readily distribute any excess heat. We have added the corresponding temperature data in S3 Fig.

*3. The author point that they demonstrate the applicability of their method to diagnose asthma. I disagree to assess a diagnostics tools a stronger study with a cohort of patients and a sound experimental plan is needed. This is beyond the scope of the present paper. Here the author discuss the comparison between a sample from one ‘asthmatic’ donor and another sample from a ‘non-asthmatic’ : this is a single comparison experiment (Fig 7). I would suggest the author to perform repetability and reproducibility experiments to ensure that the difference found can serve as a proper signal for a diagnostic tool.*

The reviewer assumes that we have used one sample only per group (asthmatic vs. non-asthmatic); however, we had 5 asthmatic donors (both sexes, between 25 and 80 years old, allergic and non-allergic asthma) and compared the migration speeds to 6 non-asthmatic donors. This was not clear in our manuscript and has been clarified now (pp 20 in the manuscript). While N=5 is still far from a clinical study, it demonstrates that our finding of differences in the migration patterns between asthmatics and non-asthmatics are reproducible. Also, they are in line with the results of Sackmann et al. (https://doi.org/10.1073/pnas.1324043111) and expand on them (using IL-8 in addition to fMLP).

Reviewer #2

*1. I didn’t get the motivations for measuring neutrophil migration speed under different control experiments. I am wondering if authors could explain more about the motivation and what kind of hints that could be biologically meaningful as a simple take-away message or future directions.*

This question can be understood as either asking for a general motivation why neutrophils have been chosen as an object of investigation and why so many different conditions have been examined; or as a question about a motivation for different control experiments. In the latter case, it is because the neutrophils came from different donors and were measured as soon as possible after blood extraction but after a certain (variable) preparation time (isolation of cells, setup of the tracking experiment). To account for variations of the donor and preparation speed, we always started with a new control experiment as an internal reference. We have added this information to the manuscript (pp. 7).

As for the general motivation of measuring neutrophils in different conditions: We have chosen neutrophils as an object for demonstrating the capabilities of our method for two reasons: (1) Neutrophils are fast movers among migrating cells and can also assume a great variety of shapes, in particular when passing through narrow pores. Therefore, they pose a challenge for cell detection and cell tracking. We reckoned that if we would be able to demonstrate that our method was capable of tracking neutrophils, it would make for a strong argument in favor of the method. (2) Neutrophil migration is an important topic in itself, because it is instrumental to understand the time course of the innate immune reaction. Also, because of that, a number of reference publications exist that our results could be compared to, which is desirable when exploring the capabilities of a new method. While comparing, we found a number of interesting parameters to look at (concentration of chemoattractant, stiffness of the matrix etc.) and measured those using our method.

*2. Authors mention using ‘minimum phase projection’ along z for extracting axial coordinates. Do you mean ‘minimum intensity projection’? Most of in-line holography can do minimum intensity projection for z axial tracking, but phase projection is not very common.*

A reconstructed hologram contains both phase shift and intensity information of the light field. We chose to work with the phase information only, because the contrast is usually stronger in the phase images. We reconstructed a virtual z-stack and constructed a minimum projection of those phase values, hence the term "minimum phase projection". We recognize now that this term is confusing, since the well-known term "minimum intensity projection" uses the word "intensity" in the sense of "pixel value" and as such can be applied to stacks of phase shift images (and any digital image stack). We amended the wording to "phase shift minimum projection".

*3. Three axes coordinates were measured but only lateral axes were used in studies. During the experiment measurements, z information was also recorded. However, when calculating average undirected speed and average velocity of all cells outward collagen droplet, only lateral coordinates were used. In authors self-explanation, it might be attributed to 20-30um axial resolution is not good enough for such a tracking purpose. But assuming migration along three axes are independent from each other, as shown in Fig 3B, the migration distance can be as large as tens of um, which is in the resolvable orders of system’s axial resolution. Since the sample is a 3D matrix, it might be very interesting to include the information from z direction, as hinted by a similar experiments, however, targeting at sperm migration where z directional migration shows unique sperm swimming patterns (Su, Ting-Wei, PNAS, 109 (40), 2012).*

We agree that using the third dimension for tracking would add value to the results. However, doing so was hindered by the fact that the 20 - 30 μm z-resolution is a lower bound only. This lower bound was determined by measuring the position of microbeads with a known z-distance in a phase shift image created by our device (see new S4 Fig for the measurement scheme). The axial position of real cells can be more ambiguous, as shown in Figs 2B and 2D. In the latter figure it can be seen that the z coordinate is rather unstable and may fluctuate sometimes across tens of millimetres in less than a minute; a type of noise which would result unrealistically high velocities and less reliable tracking when included in our computation. Still, the ensemble z-position can be computed due to the high number of cells and the random fluctuations cancelling each other out.

*4. Nonlinear response in Fig. 5A. The average unidirectional speed is quite different in different collagen concentrations. Temporal nonlinear response is expected. However, I am wondering if authors might have some additional explanations that why in both figures in Fig. 5A, the flatten or maximum of s or v is not ordered or proportional to collagen concentration, but instead, is very nonlinear. How many repetitions or collagen samples that the experiments has done? Also, it might be attributed to the conditions of sample making, like temperature, polymerization time. Similar unusual observation can also be seen in Fig. 5B, where 1mg/mL is way too higher than any other concentrations.*

The reviewer asks to explain the rather different cell migration speeds found at the lowest used collagen concentrations of 1mg/ml (cf. fig. 5 A and B). Indeed, as the reviewer suspects, we observed a strongly non-linear viscosity/stiffness of the collagen, from a rather liquid state (~1mg/ml), to a dilute gel (<5mg/ml) to a semidilute gel (>5mg/ml), which is also in line with previous reports (see Gobeaux et al.; doi:10.1039/B922151D). We discuss this phenomenon in our manuscript on p. 19: "The increased apparent mobility of the cells in the lowest collagen concentration is most likely due to the decreased stiffness of collagen, which at 1 mg/ml was at the brink of becoming a viscous liquid." This translates into a markedly different motility pattern of the neutrophils (they appear to be more like swimming in water). All tracking experiments have been carried out at least three times for every collagen concentration.

*5. During discussion part, in Line 513-520, I don’t think that the large FOV and high frame rate are the unique strength or improvement of current system, compared with other similar in-line holography. In my understanding, the difference just merely results from the hardware configuration (like just change to a large camera), without fundamental breakthrough of the technique. If author believes so, it might be more convincing to discuss more about the improvements of current apparatus compared to previous ones.*

The reviewer points out that our work does not present a "fundamental breakthrough of the technique". While this is arguably true, we add a number of smaller improvements to the state of the art of which we believe that, when taken together, make for a substantial contribution to the field. In particular, we combine (1) a larger sensor (8.6x6 mm) with a higher than usual pixel resolution (41 Mpixel) with (2) a comparatively fast framerate (0.25Hz, compare e.g. to similar work by Francois et al. (DOI 10.1126/sciadv.abf3882) who employ a framerate of 0.03 Hz, which makes it difficult to resolve the movement of fast cells like neutrophils); with (3) a useful, easy to prepare, yet readily adaptable 3D-assay (submersed collagen droplet); and (4) an important, yet challenging cell type (neutrophil granulocytes), which, for the above mentioned reasons can be hard to 3D-track using inline holography.

*6. I am also curious that why the collagen has to be droplet. Can that also be cells cultured in petri-dish or embedded in collage 3D matrix with petri-dish? I believe the latter could broader the interest of readers.*

We believe the submersed droplet assay used in our experiments to be favorable because (1) it is easy to manufacture while (2) allowing for a gradient in x-y-direction. Other arrangements are either more difficult to set up (e.g. require microfluidic channels or moulds etc) or allow for a z-gradient only (e.g. embedding neutrophils in a completely covered petri-dish). Since the lateral resolution of inline holographic setups is about one order of magnitude better than the resolution in z-direction, a x-y-gradient is preferable. Also (albeit less important) filling a whole petri-dish usually requires more cells and material to analyze.

*7. I would recommend authors to do more statistical analysis of the data to show the significant difference between different migration patterns. This will make the scientific story more convincing.*

The reviewer asks for more statistical analysis of our data to prove the significance of the differences found in the migration patterns of neutrophils. We understand this request as pertaining particularly to the comparison of neutrophil migration patterns of asthmatics vs. non-asthmatics. We have now done a statistical analysis of these data by looking at the distributions of the outward velocities (directed chemotactic velocitiy) of stimulated vs. unstimulated neutrophils for both chemoattractants (IL-8 and fMLP). In particular, we compared 75 outward-velocity values after 2.5min (i.e. 5 minutes of tracking, leaving out the onset of migration, see. fig. 7 A and B) using twelve different experiments (3 donors for asthma-condition and 3 for non-asthma condition vs. the respective control groups). The result of these comparisons are displayed in S7 Fig. It can be seen that IL-8 might be better suited than fMLP for telling apart asthma from non-asthma-conditions (p-values of 0.03* and 0.09 for IL-8 and fMLP, respectively). However, we believe that this analysis should be taken with caution because a thorough statistical analysis would require a much larger cohort of individuals for both groups. In this work, we merely attempted to replicate the results of Sackmann et al. ( https://doi.org/10.1073/pnas.1324043111 ) with our holographic setup.

Reviewer #3

*1. A common problem of collagen is the formation of bubbles during solidification (see Fig5 PMC7757667). Is it possible to recognize bubbles in the min projections and eventually decide to discard the sample? Or is it suggested to check also the diffraction images or inspect sample with another method before imaging?*

We had some minor initial problems with bubbles; but they can be mostly avoided by careful, slow pipetting of the collagen droplet. In rare cases, bubbles occured on top of the medium (as opposed to bubbles in the collagen). In such cases, the concerned areas have been completely removed prior to the subsequent tracking. We reckon, that the reviewer wanted to know if this removal could be done automatically. While we think that this might be feasible, we have inspected our diffraction data prior to reconstruction and tracking by eye. // The bubbles in the images of the referred paper (Fig5 PMC7757667) are most likely due to the closed system, which makes the removal of bubbles much harder than in the open system used by us.

*2. Not clear if the expected cell diameter should be known for digital reconstruction. Please also explain if the system can be used to image together cell types of different size (i.e. neutrophils and dendritic cells).*

Knowing the exact cell diameter is not necessary for our method to work; it suffices to know the order of magnitude (e.g. tens of micrometers as opposed to a few micrometers only). However, as the digital reconstruction is done in slices, the slice thickness should be chosen such that a good compromise between precision (smaller thickness, more slices) and computing power (less but thicker slices) is made. We have changed our text accordingly (p. 9, paragraph 1) to motivate, why we have chosen 10 μm as the thickness of our slices. Average neutrophil diameter is cited from a newly added reference (10.1152/jappl.1990.69.5.1767).

To answer the reviewers second question: It is well possible to image two similarly sized cell types in one experiment (again roughly within one order of magnitude). However, it might turn out rather difficult to tell the two cell types apart by their appearance in the holographic images. They might be told apart however, if they displayed different motion patterns (like neutrophils and dendritic cells).

*3. Fig1, a histogram with the number of cells detected at different z depths vs. the expected number could clarify how cell detection is robust to positioning along z.*

We understand the reviewer’s question such that (s)he proposes to assume a constant cell density throughout the collagen droplet and compare cell numbers in z-slices to the collagen volume ("expected number"). However, the volume of the collagen drop is not easy to determine using inline holography; only small objects with a sharp edge produce good contrast (i.e. lateral change in refraction index on a distance of the wavelength of the used light). This means that we can not see the boundary of the collagen droplet in the reconstructed slices. We only see the very edge of the droplet at the bottom because there, the above conditions to produce contrast are met. Therefore, we can not determine the volume of the slice of the collagen droplet which in turn prevents us from carrying out the reviewer’s idea to compare real cell numbers to the expected ones. Also, it is not really clear how homogeneous the cell density really is, especially when we take into account that during gelation of the collagen, some sedimentation of cells will probably occur (leading to a density gradient towards the bottom). We have, however, included additional data on the precision of our holographic method in determining the z-position of a phase object (microbeads, cf. supplemental material S4 fig.).

*4. Special care was put to create a time series of minimum projections and depth maps for neutrophils. This was achieved by relying on Gouy phase anomaly, minimum projection, and an averaging scheme that accounts for non-globular cell shapes. In L233, Fig2A it is not clear to me which is the connected foreground component. Assuming the black dots.*

Indeed the black dots in Fig. 2A represent the foreground components (cells). We have indicated this in our paragraph: "Cells were detected by thresholding the minimum projections at each time point to separate foreground pixels (value <= 80, cells) from background pixels (value > 80)." For improved clarity, we added the following sentence:

"In Fig. 2A, panel 3 shows the minimum projection and corresponding depth information. Panel 4 shows the foreground-components (white pixels) resulting from thresholding the minimum projection. For each component, its corresponding area in the depth map was considered and averaged in order to obtain a scalar z value." (pp. 11)

*5. Min proj. and depth map saved space. However, please clarify if cells lying on top of the other along z be resolved as separate objects.*

This is a good question which has interested us, too, from the start. We found that cells lying on top of each other can usually not be told apart. In most cases, the cell closer to the sensor will show a stronger phase shift and occludes the one further from the sensor in the minimum projection. Therefore, as pointed out in the manuscript, the method works for smaller cell densities only (pp 22, 2nd paragraph). It relies on the fact that at sufficiently low densities, the number of such occlusions at any given time is small in relation to the total number of cells imaged.

*6. Although the authors assessed the formation and stability of gradients within the droplet, it would be interesting to know in Fig3A if certain regions have higher motility (i.e. at the droplet boundaries due to fiber orientation?) A motility heatmap computed using optical flow (like Fig6C, PMC6881817), or maximum speed at different locations may show this.*

Indeed, we should find faster moving cells towards the rim of the droplet because this is, were the gradient has its steepest slope. We have therefore investigated the undirected cell speed along the drop radius, with inconclusive results. This is probably due to the dome-shape of the droplet. This shape causes cells at different heights (z-positions) to be close to the droplet rim at different radii (smaller radii, the higher in the droplet). Since we look at the minimum projections only, we see fast moving cells on every radius, albeit the number of such cells should decrease towards the center. This is further complicated by the fact that our method discards the speed in z-direction. For these reasons, we have not included these results in our manuscript.

*7. I recommend depositing raw data in an open bioimage repository, if possible. Diffraction images can be useful to others developing computational methods for reconstruction, while reconstructed videos can be helpful to develop analysis tools.*

This is a good point. Once our manuscript has been accepted, we plan to upload a complete set of holographic raw data to the image data resource (IDR, https://idr.openmicroscopy.org/).

*8. L78 missing reference*

We have now inserted the missing reference.
